# Perceptual formant discrimination during speech movement planning

**DOI:** 10.1371/journal.pone.0301514

**Published:** 2024-04-02

**Authors:** Hantao Wang, Yusuf Ali, Ludo Max

**Affiliations:** Department of Speech and Hearing Sciences, University of Washington, Seattle, Washington, United States of America; National Taiwan Normal University, TAIWAN

## Abstract

Evoked potential studies have shown that speech planning modulates auditory cortical responses. The phenomenon’s functional relevance is unknown. We tested whether, during this time window of cortical auditory modulation, there is an effect on speakers’ perceptual sensitivity for vowel formant discrimination. Participants made same/different judgments for pairs of stimuli consisting of a pre-recorded, self-produced vowel and a formant-shifted version of the same production. Stimuli were presented prior to a “go” signal for speaking, prior to passive listening, and during silent reading. The formant discrimination stimulus /uh/ was tested with a congruent productions list (words with /uh/) and an incongruent productions list (words without /uh/). Logistic curves were fitted to participants’ responses, and the just-noticeable difference (JND) served as a measure of discrimination sensitivity. We found a statistically significant effect of condition (worst discrimination before speaking) without congruency effect. Post-hoc pairwise comparisons revealed that JND was significantly greater before speaking than during silent reading. Thus, formant discrimination sensitivity was reduced during speech planning regardless of the congruence between discrimination stimulus and predicted acoustic consequences of the planned speech movements. This finding may inform ongoing efforts to determine the functional relevance of the previously reported modulation of auditory processing during speech planning.

## Introduction

Behavioral and neurophysiological studies of both limb and speech movements consistently show that sensory processing is modulated during active movements [1, 2]. Such movement-induced sensory modulation is commonly interpreted in the framework of predictive motor control, in which the sensorimotor system uses the predicted sensory consequences of a motor command to generate and control the movement [3–5].

For example, using extracranial neural recording techniques such as electroencephalography (EEG) and magnetoencephalography (MEG), many studies with human participants have shown attenuated brain responses to either movement-generated sensory feedback [6–9] or external sensory stimuli delivered during movement [6, 10, 11]. On the other hand, intracranial recordings in both humans and animal models have revealed a more complex picture, with vocalization or limb movements leading to both suppression and facilitation across different neuronal populations or single cells in auditory or somatosensory cortex [12–18]. At the behavioral level, many studies have found that limb movements lead to an attenuated perception of self-generated tactile, auditory or visual stimuli, including increased perceptual thresholds and decreased sensitivity [19–23]. However, several recent studies have shown that movement modulates perception in a complex manner. For example, as compared with externally generated stimuli, the perception of self-generated sensory feedback was found to be enhanced at low stimulus intensity but attenuated at high stimulus intensity [24, 25], or enhanced at an early time point and attenuated at a later time point [26, 27].

In order to fully understand how active movements affect sensory processing and perception, one also needs to consider that the auditory and somatosensory systems are already modulated during vocalization and limb movement planning prior to movement onset [13, 18, 28–32]. Here, we address this topic further in the context of our laboratory’s series of speech studies that compared long-latency auditory evoked potentials (AEPs) elicited by pure tone probe stimuli delivered during the planning phase in a delayed-response speaking task versus control conditions without speaking. The probe tone stimulus was always delivered 400 ms after initial presentation of a word and 200 ms prior to the *go* signal cueing over production (or at the equivalent time point in the passive listening and/or silent reading control conditions). Results consistently indicated that the amplitude of the cortical N1 component in the AEPs is reduced prior to speaking [32–35]. However, the functional relevance of this pre-speech auditory modulation (PSAM) phenomenon remains entirely unknown.

To date, only Merrikhi and colleagues [36] have investigated potential perceptual correlates of PSAM. Adapting the delayed-response tasks used in our previous PSAM studies, they asked participants to compare the intensity of two pure tone stimuli in both speaking and no-speaking conditions. The *standard* stimulus with a fixed intensity level was presented at the beginning of each trial. The *comparison* stimulus with varying intensity was presented during the speech planning phase at the time point where PSAM had been previously demonstrated. Based on a two-interval forced choice intensity discrimination test (“Which one was louder?”), the speaking condition showed (a) a statistically significantly higher point of subjective equality (i.e., the comparison stimulus had to be louder to be perceived equally loud as the standard stimulus), and (b) statistically significantly lower slope values for the psychometric functions (i.e., greater uncertainty in the perceptual judgments). Thus, results were consistent with the idea that PSAM during speech movement planning is associated with an attenuation in the perception of auditory input. However, given that [36] tested only intensity perception and only used pure tones, it remains to be determined whether speech planning modulates other perceptual processes with more direct relevance for monitoring auditory feedback once speech is initiated. For example, it is possible that prior to speech onset neuronal populations with different characteristics are already selectively inhibited and facilitated to suppress the processing of irrelevant events but enhance the processing of speech-related auditory feedback. In addition, it is not clear to what extent the results of [36] may have been influenced by a working memory component. In their paradigm, each trial’s time interval between the standard and comparison stimuli was 900 ms, and the target word to be spoken, read, or listened to by the participant always appeared on a computer monitor during this comparison interval.

To further investigate the functional relevance of PSAM, the current study combined our prior delayed-response speaking task paradigm with a novel same/different formant discrimination perceptual test that used brief stimuli derived from the participant’s own speech and delivered as a pair centered around the exact time point where PSAM has been previously demonstrated [32–35, 37]. The first two formants, or resonance frequencies of the vocal tract, are critical for the production of vowels, and speakers are sensitive to formant frequency changes perceived in their auditory feedback [38–43]. We therefore asked participants to make the same/different judgments for comparison stimuli that were created by extracting and truncating a pre-recorded self-produced vowel, and digitally altering the formant frequencies of the second stimulus. We controlled the congruency between these stimuli for the formant discrimination test and the vowel of the words in the delayed-response speaking task by using two different word lists: one list only included words containing the same vowel as the discrimination stimuli and the other list excluded words containing that vowel. Silent reading (Reading condition) and passive listening (Listening condition) were included as control conditions. We hypothesized that, if auditory feedback monitoring starts being suppressed during the speech planning phase, formant discrimination sensitivity would decrease in the Speaking condition as compared with the control conditions. Alternatively, if the auditory system is selectively tuned to the predicted acoustic outcomes of the planned speech movements, formant discrimination in the Speaking condition may be enhanced, especially when the discrimination stimuli are congruent with the vowel in the predicted acoustic outcome (i.e., planned production).

## Materials and methods

### Participants

Twenty-six right-handed adult native speakers of American English (16 women, 10 men, age *M* = 22.90 years, *SD* = 4.68 years, range = 18–36 years) with no self-reported history of speech, hearing, or neurological disorders participated between 07/07/2021 and 07/08/2022. Based on a pure tone hearing screening, all participants had monaural thresholds at or below 20 dB HL at all octave frequencies from 250 Hz to 8 kHz in both ears. All participants provided written informed consent, and all procedures were approved by the Institutional Review Board at the University of Washington.

### Instrumentation

Inside a sound-attenuated room, participants were seated approximately 1.5 m from a 23-inch monitor. Their speech was captured by a microphone (WL185, Shure Incorporated, Niles, IL) placed 15 cm from the mouth and connected to an audio interface (RME Babyface Pro, RME, Haimhausen, Germany). The audio interface was connected to a computer with custom software written in MATLAB (The MathWorks, Natick, MA, United States) that recorded the speech signal to computer hard disk. The output of the audio interface was amplified (HeadAmp6 Pro, ART ProAudio, Niagara Falls, NY) and played back to the participant via insert earphones (ER-1, Etymotic Research Inc., Grove Village, IL), providing speech auditory feedback throughout the whole experiment. In addition, the insert earphones were also used to deliver the binaural auditory stimuli for formant discrimination testing and playback of the participant’s previously recorded speech in the Listening condition (see below).

Before each recording session, the settings on the audio interface and the headphones amplifier were adjusted such that speech input with an intensity of 75 dB SPL at the microphone resulted in 73 dB SPL output in the earphones [44]. To calibrate the intensity of the speech signal in the earphones, a 2 cc coupler (Type 4946, Bruel & Kjaer Inc., Norcross, GA) was connected to a sound level meter (Type 2250A Hand Held Analyzer with Type 4947 ½″ Pressure Field Microphone, Bruel & Kjaer Inc., Norcross, GA).

### Procedure

The experiment consisted of two parts, a pre-test to record the participant’s productions to be used for the creation of the auditory stimuli for formant discrimination testing, and a series of speaking, listening, and silent reading tasks during which formant discrimination was tested. The pre-test consisted of thirty trials of a speech production task. During each trial, the word “tuck” appeared in green color on a black background and remained visible for 1500 ms. The participant spoke the word when it appeared. After the pre-test was completed, the experimenter used a custom MATLAB script to examine the thirty productions of “tuck” offline and manually mark the onset and offset of the vowel /uh/ (International Phonetic Alphabet symbol /ʌ/) for each trial by visually inspecting the waveform and a wide-band spectrogram. The MATLAB script then extracted the frequencies of the first two formants (F1 and F2) of the middle 20% of each production (a window from 40% to 60% into the vowel duration) as tracked by the Audapter software using the linear predictive coding algorithm [45, 46]. The median F1 and F2 frequencies of the thirty trials were calculated and the pre-test token closest to the median F1 and F2 was selected based on Euclidean distance in the F1-F2 space. The middle 60 ms of the vowel in the selected token was then used to generate the stimuli for formant discrimination testing. Truncated vowels were used so that the two auditory stimuli could be presented back-to-back as close as possible to the time point where PSAM had been demonstrated in our previous studies. Each participant’s chosen production was first modified with a linear amplitude envelope to create a 10 ms onset rise and 10 ms offset fall. Next, eleven formant-shifted versions of this truncated vowel were created with the Audapter software by shifting both F1 and F2 upward from 0 to 250 cents in 25 cents increments (i.e., 0 cents, +25 cents, +50 cents, etc.; note that 1200 cents = 12 semitones = 1 octave). To control for any unwanted effects caused by processing in the Audapter software, the processed version with 0-cent shift was used as the standard syllable in the formant discrimination test instead of the original truncated syllable.

The main part of the study included three conditions (Speaking, Listening, and Reading) with two different word lists. Thus, each participant completed six tasks. The order of the conditions and the word lists were randomized for each participant, but within the same word list, the Listening condition always had to be completed after the Speaking condition as the participant’s own recorded speech had to be played back in the Listening condition.

**Fig 1:**
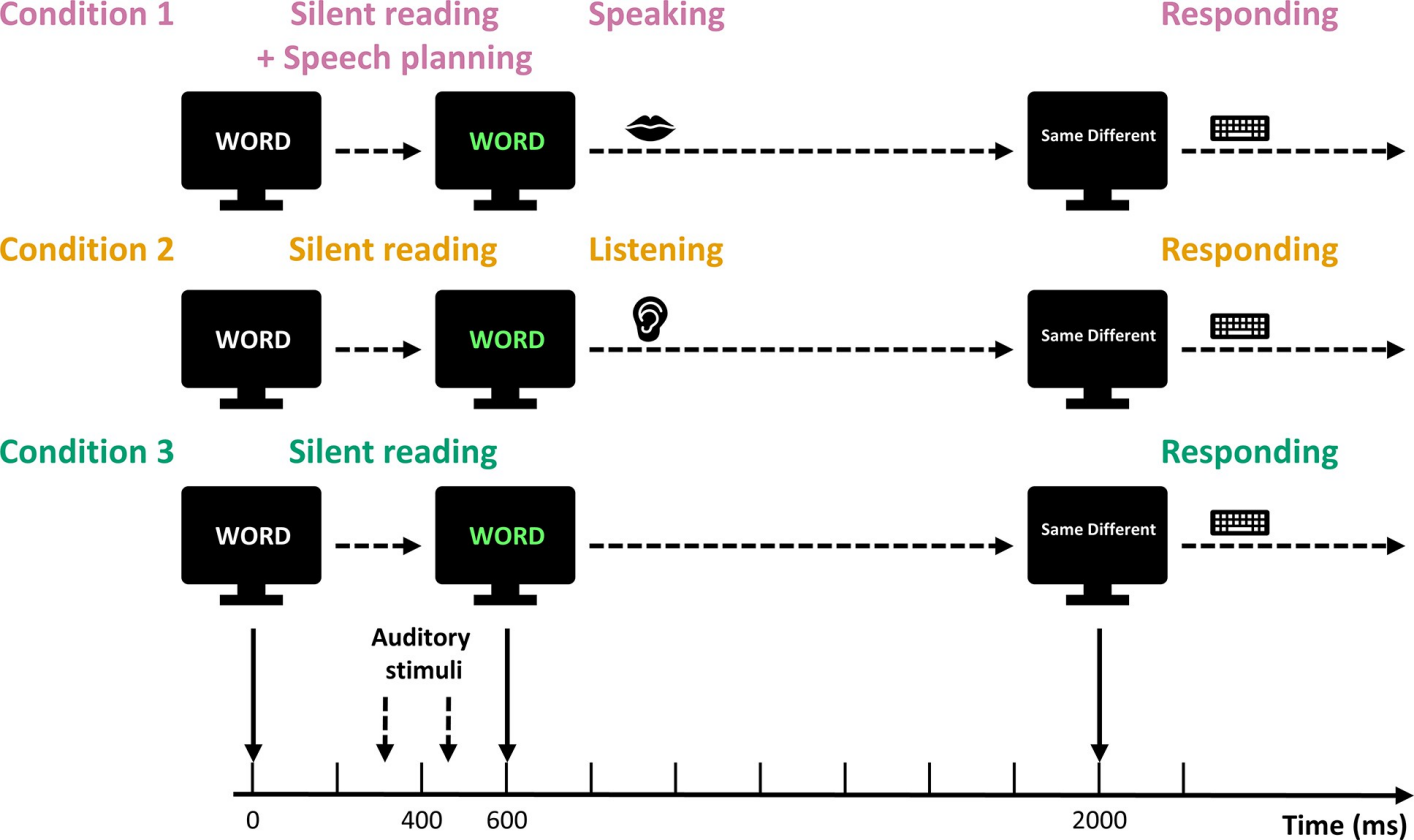
Experimental procedure. Each trial started with a white word on a black screen. During the white word period, two truncated vowels, a standard stimulus (at 290 ms) and a comparison stimulus (at 450 ms), were played to the participants. The word changed to green at 600 ms and this color change served as the go signal in the Speaking condition. In the Listening condition, participants listened to playback of their own production after the word changed to green. In the Reading condition, participants silently read the word. At 2000 ms, the green word disappeared and the participants were asked to judge whether the standard and comparison stimuli sounded the same or different by pressing keys on a keyboard.

Each task consisted of 110 trials. Each trial began with a white word appearing on a black background on a computer monitor (Fig 1). The word was chosen randomly from the applicable word list. The white word remained on the screen for 600 ms. After 600 ms, the color of the word on the screen changed from white to green, and this change in color served as the *go* signal in the Speaking condition. The green word stayed on the screen for 1400 ms. While the word in white characters was displayed on the monitor, the standard stimulus (0 cents shift, 60 ms duration) was first played through the earphones at 290 ms. Then, 100 ms after the end of the standard stimulus (450 ms after the white word appeared), the comparison stimulus was played. The comparison stimulus was randomly selected from the eleven shifted versions of the truncated syllable (0 to +250 cents). The two stimuli were played at ~75 dB SPL (the formant shifting technique sometimes induces a small intensity difference up to ~2 dB). The timing of the two syllable stimuli was chosen such that the pair was centered around the time point for which PSAM had been documented in our previous studies (i.e., 400 ms after presentation onset of the word in white characters and 200 ms prior to the *go* signal; [32–35, 37]).

When the green word disappeared, a prompt “Same Different” was presented on the monitor for the participant to judge whether the standard and comparison stimuli sounded the same or different by pressing either the F key with their left index finger or the J key with their right index finger, respectively, on a keyboard placed on their lap. The prompt disappeared after 1500 ms or as soon as one of the two buttons was pressed. The screen then remained blank for 1000 ms until the next trial started.

In the Speaking condition, participants were instructed to say the word on the monitor out loud after the word turned from white to green. In the Listening condition, participants listened to playback of their own production of each word shown on the monitor as recorded during a preceding Speaking condition with the same word list (albeit in different randomized order). Each word was played back with the same intensity and production latency as when it had been actively produced. In the Reading condition, participants were instructed to silently read the words on the monitor without making any articulatory movements.

Each of the two word lists contained 55 CVC words containing three to four letters. To test for a potential effect of congruency between the formant discrimination stimuli and the produced words, one word list (“word list with /uh/”) included only words that had /uh/ as their syllable nucleus (e.g., “love”, “run”) whereas the other word list (“word list without /uh/”) excluded any words with /uh/ (e.g., “talk”, “sit”). The two word lists were balanced in terms of word frequency [47] and word length.

### Data analysis

**Fig 2:**
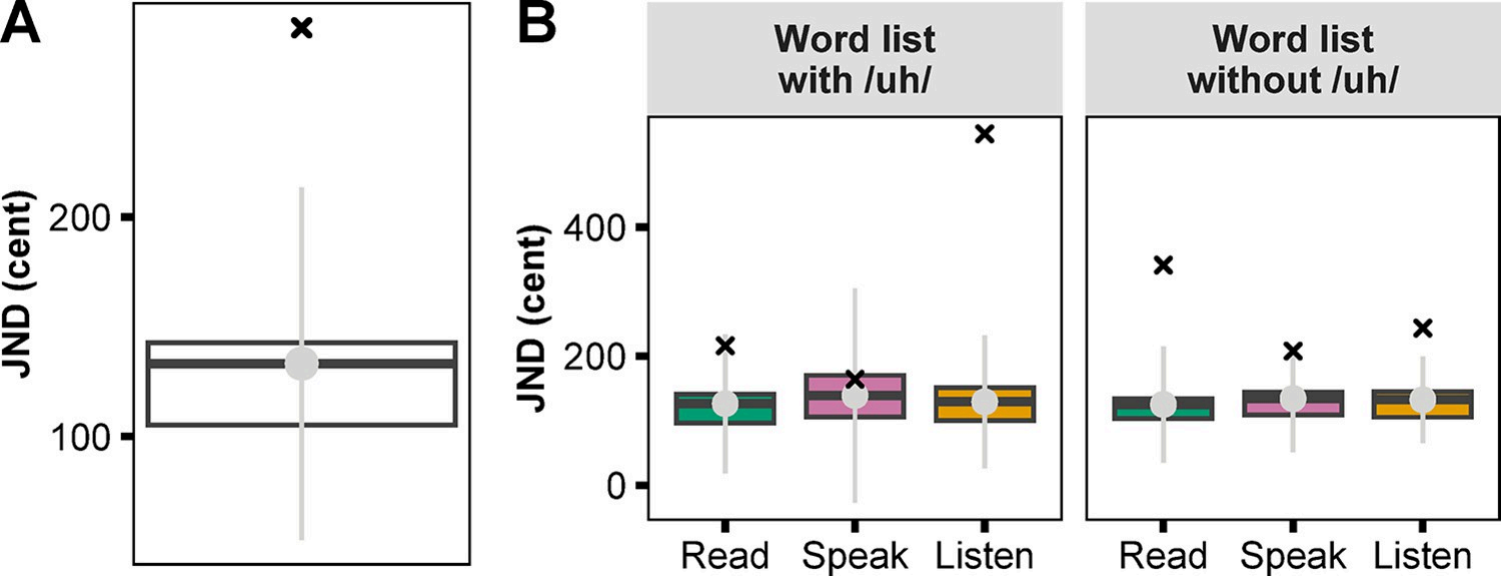
Boxplots illustrating exclusion of an outlier participant. (A) just-noticeable difference (JND) averaged across all six tasks. (B) JND by condition (Reading, Speaking, Listening) and word list. The cross symbol (×) indicates the participant who was excluded because the JND averaged across the six tasks was more than three absolute deviations (grey bars) away from the sample median (grey dots).

For each participant, a logistic regression was fitted to the formant discrimination response data from each of the six tasks using the *glm()* function in the R software [48]. Two parameters, the just-noticeable difference (JND, defined as the shift amount at which the logistic fit predicts a 50% chance of responding “Different”) and the slope of the logistic curve were calculated from each fit. The key-pressing response time for each trial was also extracted. Two steps were taken to exclude data points that were outliers. First, three participants with a negative JND or slope were excluded. Second, the sample distributions of the JND averaged across the six tasks (Fig 2A) and the JND of each task (Fig 2B) were examined. One additional participant was excluded because their JND averaged across the six tasks was more than three absolute deviations away from the sample median [49]. All data from the remaining 22 participants were included in the statistical analyses.

All statistical analyses were conducted in the R software [48]. JND, slope, and response time were used as dependent variables for which we conducted a two-way repeated measures analysis of variance (rANOVA) with Condition (Reading, Speaking, and Listening), Word list (“word list with /uh/” and “word list without /uh/”) and their interaction as within-subjects variables. To account for potential violations of the sphericity assumption, the degrees of freedom for within-subject effects were adjusted using the Huynh–Feldt correction [50]. Post-hoc tests of simple effects were conducted by means of paired *t*-tests adjusted with the Holm-Bonferroni method [51]. For effect size calculations, generalized eta-squared ($\eta_{G}^{2}$) was used for rANOVA [52] and Cohen’s *d* was used for pair-wise post-hoc tests [53]. Additionally, because the formant discrimination test was a novel test for the participants, we explored potential practice effects with one-way rANOVAs for JND, slope, and response time with Task order (1 to 6) as the within-subject variable, followed by post-hoc *t*-tests. The same adjustment method for multiple comparisons was applied. Lastly, Pearson correlation coefficients were used to examine a potential relationship between response time and either the JND or slope values.

## Results

**Fig 3:**
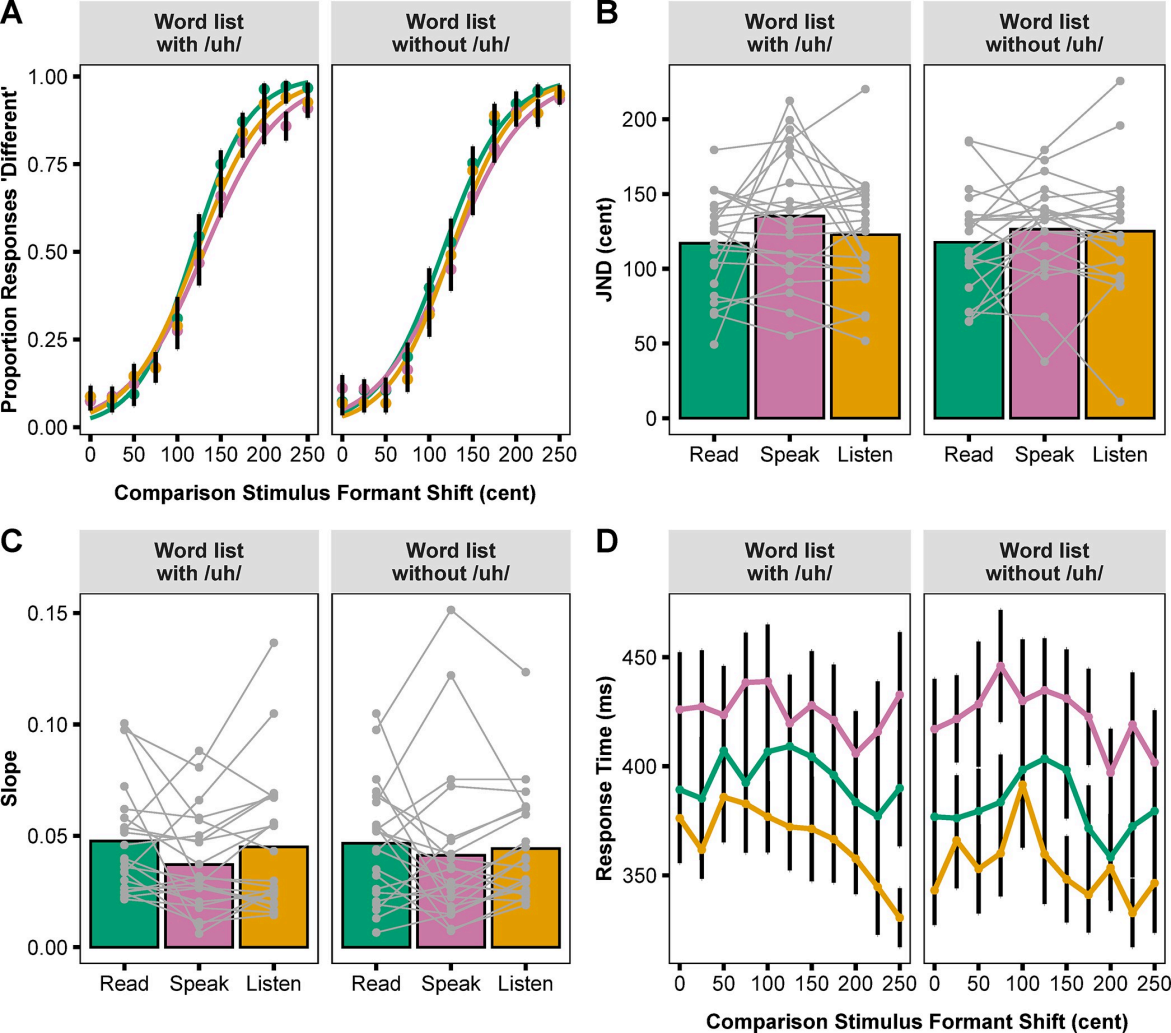
Formant discrimination task results. (A) Logistic curves fitted to group averaged data for the proportion of “Different” responses by condition and word list. (B) Mean and individual participant JNDs by condition and word list. (C) Mean and individual participant slopes by condition and word list. JND and slope were calculated from the logistic curves fitted to each participant’s responses in each task. (D) Mean keypress response time at each formant shift level of the comparison stimulus by condition and word list. Error bars correspond to standard errors.

Fig 3A shows logistic curves fitted to group averaged data for the proportion of “Different” responses at each formant shift level of the comparison stimulus in the six tasks (three conditions by two word lists). For each task, the group averaged JND and corresponding individual participant data are shown in Fig 3B. A two-way rANOVA (Conditions × Word lists) revealed that there was a statistically significant effect for Condition (*F*(2.20, 46.12) = 4.82, *p* = 0.01, $\eta_{G}^{2}$ = 0.02), but not for Word list or the interactions. Post-hoc analyses of the Condition effect revealed that the JND in the Speaking condition (*M* = 130.77 cents, *SD* = 30.29 cents) was statistically significantly larger than in the Reading condition (*M* = 117.32 cents, *SD* = 30.81 cents; *t*(21) = 3.23, *p* = 0.01, *d* = 0.69). There was no statistically significant difference in JND between the Speaking and Listening conditions (*M* = 123.91 cents, *SD* = 37.59 cents; *t*(21) = 1.56, *p* = 0.27, *d* = 0.33) or between the Reading and Listening conditions (*t*(21) = -1.49, *p* = 0.27, *d* = -0.32). A one-way rANOVA with Task order as the within-subjects effect found no statistically significant change in JND with practice (*F*(3.61, 75.82) = 1.91, *p* = 0.12, $\eta_{G}^{2}$ = 0.03).

For slope of the fitted curves, none of the main effects or interactions were found to be statistically significant (Fig 3C). However, there was a significant practice effect for slope as revealed by a Task order effect in a one-way rANOVA (*F*(4.25, 89.35) = 1.91, *p* < 0.01, $\eta_{G}^{2}$ = 0.06) and post-hoc analyses showing that slope in the first task was statistically significantly smaller than that in the fifth task (*t*(21) = -3.39, *p* = 0.04, *d* = -0.72) and the sixth task (*t*(21) = -3.38, *p* = 0.04, *d* = -0.72).

Fig 3D shows group averaged data for response time at each formant shift level of the comparison stimulus. A two-way rANOVA conducted with the response time data revealed a statistically significant main effect of Condition (*F*(1.47, 30.86) = 10.32, *p* < 0.01, $\eta_{G}^{2}$ = 0.07). Post-hoc analyses then revealed that response time in the Speaking condition (*M* = 423.88 ms, *SD* = 98.37 ms) was significantly slower than in the Listening condition (*M* = 360.22 ms, *SD* = 67.82 ms; *t*(21) = 7.39, *p* < 0.01, *d* = 1.58), but there was no significant difference between the Speaking and Reading conditions (*M* = 388.08 ms, *SD* = 87.73 ms; *t*(21) = 2.09, *p* = 0.97, *d* = 0.45) or between the Reading and Listening conditions (*t*(21) = 1.86, *p* = 0.97, *d* = 0.40). Additionally, a one-way rANOVA examining the influence of Task order revealed a statistically significant effect (*F*(2.89, 60.62) = 18.32, *p* < 0.01, $\eta_{G}^{2}$ = 0.20). Post-hoc analyses showed a significantly slower response time for the first and second task versus the third, fourth, fifth, and sixth task, and for the third, fourth and fifth task versus the sixth task (*p* < 0.05 in all pairwise comparisons).

Lastly, we calculated Pearson correlation coefficients for the relationship between response time and JND or slope in all three conditions. No statistically significant correlations were found between response time and JND. For slope, there was a significant negative correlation between response time and slope only in the Speaking condition (*r* = −0.56, *p* < 0.01; *p* > 0.07 for all other correlations).

### Discussion

Building upon previous findings of modulated AEPs and weakened intensity discrimination of pure tone stimuli during the speech planning phase, the current study examined whether speech planning modulates speakers’ ability to detect small formant frequency differences in recordings of their own vowel productions. The premise was that formant discrimination is critical for auditory feedback monitoring during speech, and that the previously documented phenomenon of PSAM [32–35, 37] may reflect either suppression or selective tuning of auditory neuronal populations in preparation for this feedback monitoring.

Participants performed same/different formant judgments for recordings of self-produced vowels during the speech planning phase before speaking (Speaking condition) as well as prior to passive listening (Listening condition) and during silent reading (Reading condition). We also examined whether congruency between the formant discrimination stimuli and the planned production would affect participants’ judgments. Logistic regression functions were fitted to the participants’ responses in each condition for both incongruent and congruent word lists. JND was calculated as a measure of formant discrimination sensitivity. We found that participants showed a small but statistically significant decrease in formant discrimination sensitivity (i.e., higher JND) during the speech planning phase in the Speaking condition as compared with the Reading condition. Although other pair-wise comparisons showed no statistically significant differences, the group average JND for the Listening condition fell in-between those for the Speaking condition and the Reading condition. Descriptively, this ranking of JND across the conditions was more clear when the vowels presented for discrimination were congruent with the vowels to be produced, but the influence of congruency was not statistically significant (no main effect or interaction).

In addition to JND, we also determined the slope of the fitted logistic curves as another psychometric measure. Statistical tests showed no significant effects of either independent variable (Condition and Word list) on these slope measures. However, unlike JND, slope showed significant changes over time and it increased from earlier to later tasks. In other words, for slope, there was a practice effect. The interpretation of slope in a same/different discrimination paradigm is not entirely straightforward but relates to the “decisiveness” of a participant’s responses given that a greater slope value indicates a more abrupt transition from “same” responses to “different” responses regardless of the JND value. Keypress response times also exhibited a significant practice effect and decreased from earlier to later trials. Thus, our slope and response time measures indicate that participants’ formant discrimination responses became more decisive and faster throughout the experiment, but these behavioral changes did not affect the JND data.

Overall, our results are mostly consistent with those of the only other study that already investigated perceptual correlates of PSAM. Merrikhi and colleagues [36] found that speech planning led to higher discrimination thresholds and higher perceptual uncertainty in a *pure tone intensity* discrimination test. Nevertheless, there are some differences between the results of the two studies. In the current study, the small decrease in formant discrimination sensitivity was only statistically significant for Speaking versus Reading, but not for Speaking versus Listening and also not for Listening versus Reading. On the other hand, in [36], the Speaking condition showed a significantly higher discrimination threshold than both the Reading and Listening conditions. Additionally, we found no effect of condition or word list on the slope of the logistic regression functions as a measure of perceptual uncertainty, whereas [36] found that perceptual uncertainty in their Speaking condition was significantly higher than in their Reading condition.

What may account for these discrepancies between the results from the two studies? First, in both studies the changes in discrimination ability observed during speech planning are very small, and, thus, participant sampling and inter-individual variability may cause inconsistency in terms of whether or not these effects reach statistical significance in a given study. Second, there is prior evidence that speech planning has different effects on the auditory processing of pure tones versus truncated syllables: although the modulation of N1 amplitude seems equivalent for the two types of stimuli, modulation of P2 amplitude, reflecting later stages of auditory processing, may be specific to speech stimuli [32]. Sensory prediction of the speech auditory input in our Listening condition may have a small modulating effect on formant discrimination, thereby reducing the difference in discrimination ability between the Speaking and Listening conditions. Third, the timing of the stimuli for comparison differed between the two studies. In [36], two 50 ms pure tones were separated by 900 ms. In the current study, two 60 ms truncated syllables were separated by only 100 ms such that both tokens could be presented as close as possible to the time point for which PSAM has been documented. It is possible that our paradigm with such a short interstimulus interval made formant discrimination overall more difficult or more variable.

Taken together, the slightly decreased formant discrimination sensitivity during the speech planning phase as compared with during silent reading and the lack of a word list congruency effect are largely consistent with a general auditory attenuation account of PSAM. The difference in formant discrimination between our speaking and listening conditions was not statistically significant, but this result is in keeping with one of our prior EEG studies demonstrating that modulation of the auditory N1 component was also observed before both speaking and listening to prerecorded versions of one’s own speech [33]. Analogous auditory predictions may be generated when planning to speak and when expecting to hear, with predictable timing, playback of the same words. Similarly, the absence of a word list effect is not entirely surprising as our previous EEG studies on PSAM have demonstrated that the effect occurs even for a pure tone (a stimulus that clearly lacks congruency with any auditory predictions generated during speech planning) and that the effect PSAM of the N1 component does not differ when the probe stimuli are pure tones versus speech syllables [32].

These results then suggest that a speaking-induced general attenuation of the auditory system already starts during the speech planning phase prior to movement onset, regardless of the acoustic similarity between the auditory input and the predicted acoustic outcomes of the planned speech movements. Evidence from several other lines of human and nonhuman vocalization studies indicates that, during the actual production, some of the suppressed auditory neurons then respond more strongly when a mismatch is detected between perceived and predicted feedback [6, 13, 54–60].

Nevertheless, a number of alternative interpretations cannot be ruled out at this time. For example, it has been argued that tasks requiring discrimination of same or different syllable pairs recruit sensorimotor networks that are also involved in speech production [61, 62]. This raises the possibility that the requisite activation of these networks during the planning phase in our Speaking condition negatively impacted their contributions to the detection of subtle differences between the discrimination stimuli that were presented during the same time window. In fact, as a more narrow version of this hypothesis suggesting “interference” between sensorimotor processing during speech planning and auditory processing, PSAM may reflect neither a purposeful general suppression nor a fine-tuning of auditory cortex to optimize feedback monitoring but an active involvement of auditory neuronal populations in *feedforward* speech planning. This novel hypothesis certainly is testable, most directly with experimental paradigms examining whether individual participant PSAM measures relate more closely to aspects of speech that reflect the extent of feedforward preparation or, alternatively, that reflect the implementation of feedback-based corrections.

In sum, the current study examined perceptual correlates of PSAM by investigating participants’ formant discrimination ability prior to speaking, prior to passive listening, and during silent reading. We found that speech planning led to a small but statistically significant decrease in formant discrimination sensitivity in the absence of a statistically significant effect of congruency between the discrimination stimuli and the predicted acoustic outcomes of the planned speech movements. This work provides new behavioral evidence regarding modulation of the auditory system during speech movement planning and motivates further research into the phenomenon’s functional relevance.

## References

[1] Press C, Kok P, Yon D. The Perceptual Prediction Paradox. Trends in Cognitive Sciences. Elsevier Ltd; 2020. pp. 13–24. doi: 10.1016/j.tics.2019.11.003 31787500

[2] Waszak F, Cardoso-Leite P, Hughes G. Action effect anticipation: Neurophysiological basis and functional consequences. Neuroscience and Biobehavioral Reviews. Pergamon; 2012. pp. 943–959. doi: 10.1016/j.neubiorev.2011.11.004 22108008

[3] Miall RC, Wolpert DM. Forward models for physiological motor control. Neural Networks. 1996;9: 1265–1279. doi: 10.1016/s0893-6080(96)00035-4 12662535

[4] Sperry RW. Neural basis of the spontaneous optokinetic response produced by visual inversion. J Comp Physiol Psychol. 1950;43: 482–489. doi: 10.1037/h0055479 14794830

[5] Von Holst E, Mittelstaedt H. The Principle of Reafference: Interactions Between the Central Nervous System and the Peripheral Organs. 1950.

[6] Houde JF, Nagarajan SS, Sekihara K, Merzenich MM. Modulation of the auditory cortex during speech: An MEG study. J Cogn Neurosci. 2002;14: 1125–1138. doi: 10.1162/089892902760807140 12495520

[7] Aliu SO, Houde JF, Nagarajan SS. Motor-induced suppression of the auditory cortex. J Cogn Neurosci. 2009;21: 791–802. doi: 10.1162/jocn.2009.21055 18593265 PMC2944400

[8] Hughes G, Waszak F. ERP correlates of action effect prediction and visual sensory attenuation in voluntary action. Neuroimage. 2011;56: 1632–1640. doi: 10.1016/j.neuroimage.2011.02.057 21352924

[9] Bäß P, Jacobsen T, Schröger E. Suppression of the auditory N1 event-related potential component with unpredictable self-initiated tones: Evidence for internal forward models with dynamic stimulation. International Journal of Psychophysiology. 2008;70: 137–143. doi: 10.1016/j.ijpsycho.2008.06.005 18627782

[10] Numminen J, Curio G. Differential effects of overt, covert and replayed speech on vowel-evoked responses of the human auditory cortex. Neurosci Lett. 1999;272: 29–32. doi: 10.1016/s0304-3940(99)00573-x 10507535

[11] Ito T, Ohashi H, Gracco VL. Changes of orofacial somatosensory attenuation during speech production. Neurosci Lett. 2020;730. doi: 10.1016/j.neulet.2020.135045 32413541

[12] Suga N, Shimozawa T. Site of neural attenuation of responses to self-vocalized sounds in echolocating bats. Science. 1974;183: 1211–1213. doi: 10.1126/science.183.4130.1211 4812353

[13] Eliades SJ, Wang X. Corollary Discharge Mechanisms During Vocal Production in Marmoset Monkeys. Biol Psychiatry Cogn Neurosci Neuroimaging. 2019;4: 805–812. doi: 10.1016/j.bpsc.2019.06.008 31420219 PMC6733626

[14] Creutzfeldt O, Ojemann G, Lettich E. Neuronal activity in the human lateral temporal lobe. II. Responses to the subjects own voice. Exp Brain Res. 1989;77: 476–489. doi: 10.1007/BF00249601 2806442

[15] Greenlee JDW, Behroozmand R, Larson CR, Jackson AW, Chen F, Hansen DR, et al. Sensory-motor interactions for vocal pitch monitoring in non-primary human auditory cortex. PLoS One. 2013;8. doi: 10.1371/journal.pone.0060783 23577157 PMC3620048

[16] Müller-Preuss P, Ploog D. Inhibition of auditory cortical neurons during phonation. Brain Res. 1981;215: 61–76. doi: 10.1016/0006-8993(81)90491-1 7260601

[17] Singla S, Dempsey C, Warren R, Enikolopov AG, Sawtell NB. A cerebellum-like circuit in the auditory system cancels responses to self-generated sounds. Nat Neurosci. 2017;20: 943–950. doi: 10.1038/nn.4567 28530663 PMC5525154

[18] Eliades SJ, Wang X. Sensory-motor interaction in the primate auditory cortex during self-initiated vocalizations. J Neurophysiol. 2003;89: 2194–2207. doi: 10.1152/jn.00627.2002 12612021

[19] Blakemore SJ, Wolpert DM, Frith CD. Central cancellation of self-produced tickle sensation. Nat Neurosci. 1998;1: 635–640. doi: 10.1038/2870 10196573

[20] Shergill SS, Bays PH, Frith CD, Wolpert DM. Two eyes for an eye: The neuroscience of force escalation. Science (1979). 2003;301: 187. doi: 10.1126/science.1085327 12855800

[21] Bays PM, Wolpert DM, Flanagan JR. Perception of the consequences of self-action is temporally tuned and event driven. Current Biology. 2005;15: 1125–1128. doi: 10.1016/j.cub.2005.05.023 15964278

[22] Sato A. Action observation modulates auditory perception of the consequence of others’ actions. Conscious Cogn. 2008;17: 1219–1227. doi: 10.1016/j.concog.2008.01.003 18299207

[23] Cardoso-Leite P, Mamassian P, Schütz-Bosbach S, Waszak F. A new look at sensory attenuation: Action-effect anticipation affects sensitivity, not response bias. Psychol Sci. 2010;21: 1740–1745. doi: 10.1177/0956797610389187 21119181

[24] Paraskevoudi N, SanMiguel I. Self-generation and sound intensity interactively modulate perceptual bias, but not perceptual sensitivity. Scientific Reports 2021 11:1. 2021;11: 1–13. doi: 10.1038/s41598-021-96346-z 34429453 PMC8385100

[25] Reznik D, Henkin Y, Levy O, Mukamel R. Perceived loudness of self-generated sounds is differentially modified by expected sound intensity. PLoS One. 2015;10. doi: 10.1371/journal.pone.0127651 25992603 PMC4436370

[26] Yon D, Gilbert SJ, de Lange FP, Press C. Action sharpens sensory representations of expected outcomes. Nat Commun. 2018;9. doi: 10.1038/s41467-018-06752-7 30327503 PMC6191413

[27] Yon D, Press C. Predicted action consequences are perceptually facilitated before cancellation. J Exp Psychol Hum Percept Perform. 2017;43: 1073–1083. doi: 10.1037/xhp0000385 28263639

[28] Williams SR, Shenasa J, Chapman CE. Time course and magnitude of movement-related gating of tactile detection in humans. I. Importance of stimulus location. J Neurophysiol. 1998;79: 947–963. doi: 10.1152/jn.1998.79.2.947 9463455

[29] Eliades SJ, Wang X. Dynamics of auditory-vocal interaction in monkey auditory cortex. Cerebral Cortex. 2005;15: 1510–1523. doi: 10.1093/cercor/bhi030 15689521

[30] Seki K, Perlmutter SI, Fetz EE. Sensory input to primate spinal cord is presynaptically inhibited during voluntary movement. Nat Neurosci. 2003;6: 1309–1316. doi: 10.1038/nn1154 14625555

[31] Mock JR, Foundas AL, Golob EJ. Modulation of sensory and motor cortex activity during speech preparation. European Journal of Neuroscience. 2011;33: 1001–1011. doi: 10.1111/j.1460-9568.2010.07585.x 21261757

[32] Daliri A, Max L. Modulation of Auditory Responses to Speech vs. Nonspeech Stimuli during Speech Movement Planning. Front Hum Neurosci. 2016;10: 234. doi: 10.3389/fnhum.2016.00234 27242494 PMC4870268

[33] Daliri A, Max L. Electrophysiological evidence for a general auditory prediction deficit in adults who stutter. Brain Lang. 2015;150: 37–44. doi: 10.1016/j.bandl.2015.08.008 26335995 PMC4663101

[34] Daliri A, Max L. Modulation of auditory processing during speech movement planning is limited in adults who stutter. Brain Lang. 2015;143: 59–68. doi: 10.1016/j.bandl.2015.03.002 25796060 PMC4380808

[35] Max L, Daliri A. Limited Pre-Speech Auditory Modulation in Individuals Who Stutter: Data and Hypotheses. Journal of Speech, Language, and Hearing Research. 2019;62: 3071–3084. doi: 10.1044/2019_JSLHR-S-CSMC7-18-0358 31465711 PMC6813031

[36] Merrikhi Y, Ebrahimpour R, Daliri A. Perceptual manifestations of auditory modulation during speech planning. Exp Brain Res. 2018;236: 1963–1969. doi: 10.1007/s00221-018-5278-3 29713756

[37] Daliri A, Max L. Stuttering adults’ lack of pre-speech auditory modulation normalizes when speaking with delayed auditory feedback. Cortex. 2018;99: 55–68. doi: 10.1016/j.cortex.2017.10.019 29169049 PMC5801108

[38] Tourville JA, Reilly KJ, Guenther FH. Neural mechanisms underlying auditory feedback control of speech. Neuroimage. 2008;39: 1429–1443. doi: 10.1016/j.neuroimage.2007.09.054 18035557 PMC3658624

[39] Niziolek CA, Guenther FH. Vowel category boundaries enhance cortical and behavioral responses to speech feedback alterations. Journal of Neuroscience. 2013;33: 12090–12098. doi: 10.1523/JNEUROSCI.1008-13.2013 23864694 PMC3713738

[40] Wang H, Max L. Inter-Trial Formant Variability in Speech Production Is Actively Controlled but Does Not Affect Subsequent Adaptation to a Predictable Formant Perturbation. Front Hum Neurosci. 2022;16. doi: 10.3389/fnhum.2022.890065 35874163 PMC9300893

[41] Houde JF, Jordan MI. Sensorimotor adaptation in speech production. Science (1979). 1998;279: 1213–1216. doi: 10.1126/science.279.5354.1213 9469813

[42] Purcell DW, Munhall KG. Compensation following real-time manipulation of formants in isolated vowels. Citation: The Journal of the Acoustical Society of America. 2006;119: 2288. doi: 10.1121/1.2173514 16642842

[43] Purcell DW, Munhall KG. Adaptive control of vowel formant frequency: Evidence from real-time formant manipulation. J Acoust Soc Am. 2006;120: 966–977. doi: 10.1121/1.2217714 16938984

[44] Cornelisse LE, Gagne JP, Seewald RC. Ear level recordings of the long-term average spectrum of speech. Ear Hear. 1991;12: 47–54. doi: 10.1097/00003446-199102000-00006 2026288

[45] Cai S, Boucek M, Ghosh S, Guenther F, Perkell J. A System for Online Dynamic Perturbation of Formant Trajectories and Results from Perturbations of the Mandarin Triphthong /iau/. undefined. 2008.

[46] Tourville JA, Cai S, Guenther F. Exploring auditory-motor interactions in normal and disordered speech. Proceedings of Meetings on Acoustics. 2013;19: 060180. doi: 10.1121/1.4800684

[47] Brysbaert M, New B. Moving beyond Kučera and Francis: A critical evaluation of current word frequency norms and the introduction of a new and improved word frequency measure for American English. Behav Res Methods. 2009;41: 977–990. doi: 10.3758/BRM.41.4.977 19897807

[48] R Core Team. R: A language and environment for statistical computing. Vienna, Austria: R Foundation for Statistical Computing; 2019. Available: https://www.R-project.org/

[49] Leys C, Ley C, Klein O, Bernard P, Licata L. Detecting outliers: Do not use standard deviation around the mean, use absolute deviation around the median. J Exp Soc Psychol. 2013;49: 764–766. doi: 10.1016/J.JESP.2013.03.013

[50] Max L, Onghena P. Some Issues in the Statistical Analysis of Completely Randomized and Repeated Measures Designs for Speech, Language, and Hearing Research. Journal of Speech, Language, and Hearing Research. 1999;42: 261–270. doi: 10.1044/JSLHR.4202.261 10229445

[51] Holm S. A Simple Sequentially Rejective Multiple Test Procedure. Scandinavian Journal of Statistics. 1979;6: 65–70. Available: http://www.jstor.org/stable/4615733

[52] Olejnik S, Algina J. Generalized Eta and Omega Squared Statistics: Measures of Effect Size for Some Common Research Designs. Psychol Methods. 2003;8: 434–447. doi: 10.1037/1082-989X.8.4.434 14664681

[53] Cohen J. Statistical Power Analysis for the Behavioral Sciences. Statistical Power Analysis for the Behavioral Sciences. New York, NY: Routledge; 1988. doi: 10.4324/9780203771587

[54] Heinks-Maldonado TH, Mathalon DH, Gray M, Ford JM. Fine-tuning of auditory cortex during speech production. Psychophysiology. 2005;42: 180–190. doi: 10.1111/j.1469-8986.2005.00272.x 15787855

[55] Heinks-Maldonado TH, Nagarajan SS, Houde JF. Magnetoencephalographic evidence for a precise forward model in speech production. Neuroreport. 2006;17: 1375–1379. doi: 10.1097/01.wnr.0000233102.43526.e9 16932142 PMC4060597

[56] Behroozmand R, Karvelis L, Liu H, Larson CR. Vocalization-induced enhancement of the auditory cortex responsiveness during voice F0 feedback perturbation. Clin Neurophysiol. 2009;120: 1303–1312. doi: 10.1016/j.clinph.2009.04.022 19520602 PMC2710429

[57] Behroozmand R, Larson CR. Error-dependent modulation of speech-induced auditory suppression for pitch-shifted voice feedback. BMC Neurosci. 2011;12: 1–10. doi: 10.1186/1471-2202-12-54/FIGURES/5 21645406 PMC3120724

[58] Liu H, Meshman M, Behroozmand R, Larson CR. Differential effects of perturbation direction and magnitude on the neural processing of voice pitch feedback. Clinical Neurophysiology. 2011;122: 951–957. doi: 10.1016/j.clinph.2010.08.010 20869305 PMC3151676

[59] Eliades SJ, Wang X. Neural substrates of vocalization feedback monitoring in primate auditory cortex. Nature 2008 453:7198. 2008;453: 1102–1106. doi: 10.1038/nature06910 18454135

[60] Eliades SJ, Tsunada J. Auditory cortical activity drives feedback-dependent vocal control in marmosets. Nature Communications 2018 9:1. 2018;9: 1–13. doi: 10.1038/s41467-018-04961-8 29959315 PMC6026141

[61] Jenson D, Saltuklaroglu T. Sensorimotor contributions to working memory differ between the discrimination of Same and Different syllable pairs. Neuropsychologia. 2021;159. doi: 10.1016/j.neuropsychologia.2021.107947 34216594

[62] Jenson D, Saltuklaroglu T. Dynamic auditory contributions to error detection revealed in the discrimination of Same and Different syllable pairs. Neuropsychologia. 2022;176. doi: 10.1016/j.neuropsychologia.2022.108388 36183800

