# Supplementary material for: Perceptual formant discrimination during speech movement planning
Source: PLoS One. 2024 Apr 2;19(4):e0301514. doi: 10.1371/journal.pone.0301514 (PMC10986972; doi:10.1371/journal.pone.0301514)
Supplement: Attachment — Submitted filename: AuthorsResponesToReviewerComments.docx [file pone.0301514.s001.docx]

Authors’ response to the review of PONE-D-23-34985 “Perceptual formant discrimination during speech movement planning”

*Note: Below, all author critiques are reproduced in blue italic font. Our responses are provided in black non-italic font.*

*Reviewer #1: In this study, Wang et al. explored the impact of speech planning on speakers' perceptual sensitivity concerning vowel formant discrimination. During the task, participants were tasked with making judgments on pairs of auditory stimuli, consisting of a pre-recorded, self-produced vowel and a formant-shifted version of the same production. The findings revealed that, during the process of speech planning, subjects exhibited reduced perceptual sensitivity in the discrimination of formants, as opposed to conditions where no motor plan was engaged.*

*The manuscript demonstrates a commendable level of writing, and the analysis appears to be executed with precision. I have only a couple of inquiries, and I look forward to hearing the authors' responses.*

We thank the reviewer for these positive comments.

*I recommend that the authors conduct additional analyses as outlined below:*

*Given the framework of predictive motor control, where increased focus of the brain on the predictive process may lead to improved estimation of the sensory consequence of motor commands, thereby leading to more accurate motor outputs, it would be valuable to investigate whether there is a correlation between subjects' speech variability and perceptual sensitivity. To explore this, I suggest examining the relationship between the variability of speech output for each subject and their perceptual sensitivity. The variability of speech output can be quantified by calculating the Euclidean distance of each speech output (i.e., trial) from the centroid of the speech outputs in the F1-F2 space.*

We agree with the reviewer that the framework of predictive motor control offers many opportunities for investigations of various additional questions. We certainly also agree that one related topic in need of investigation is the role of within-speaker inter-trial variability. In fact, based on an entirely separate dataset we have a manuscript on the relation between inter-trial production variability and pre-speech auditory modulation (PSAM) in preparation for a different journal. In the present work, the question being addressed is specifically whether the time point during speech preparation where we have previously observed PSAM is characterized by a change in perceptual sensitivity for formant discrimination. That is, the objective of the current study was to investigate perceptual correlates of the PSAM phenomenon.

Clearly, speech variability – albeit interesting -- was not intended to be a topic of the present study. Therefore, we did not apply any acoustic analyses to the utterances produced during the speech tasks that involved the perceptual judgments, and thus no such data are available at this time. Furthermore, it should also be noted that the design of the current study was developed for the question under investigation (i.e., perceptual correlates of PSAM) but has several disadvantages for also conducting the different study that the reviewer suggests (i.e., a study on the relationship between production variability and perceptual sensitivity). First, our study used two different word lists, one list with only one vowel and one list with several different vowels. Thus, quantifying inter-trial variability would not be straightforward. Second, the two lists of target utterances were not controlled in terms of the consonants preceding or following the vowels as consonant context was not deemed a priority for our perceptual tests of formant discrimination. Formant-based production variability analyses would inevitably be affected by consonant context and by context-specific CV and VC formant transitions.

Taking together the facts that (a) the suggested analyses of a relationship between variability and perceptual sensitivity would address different questions than those under investigation here, (b) no acoustic data were extracted that could be used for the suggested analyses, (c) the design was less than ideal for rigorous investigation of the questions suggested by the reviewer, and (d) we have a separate manuscript in preparation describing a study of the relation between inter-trial variability and PSAM, we honestly believe that those questions are better suited for separate studies and publications that are better suited to elucidate the relationship between speech variability and perceptual sensitivity (see for example Franken et al., 2017, Individual variability as a window on production-perception interactions in speech motor control. J. Acoust. Soc. Am. 142: 2007–2018).

*We can pose the following questions:*

*1. Relationship between Overall Speech Variability and JND/Reaction Time/Slope:*

*Investigate if there is a correlation between subjects’ overall speech variability and their JND, reaction time, and slope. For instance, do subjects with higher speech variability tend to exhibit higher JNDs, and vice versa? Additionally, explore the potential impact of task conditions (Speaking, Listening, and Reading) and word congruency on these relationships.*

As mentioned above, these are all interesting questions for a complete and separate study/publication. However, the absence of our own topic of investigation (PSAM) in any of these questions also makes it clear that these questions are well beyond the scope of the paper under review. We would also like to point out that answers to some of these questions are already available in work such as the above referenced study by Franken et al. (2017).

2. Comparison of More and Less Variable Trials:

Classify trials for each subject into two categories based on speech variability in the F1-F2 space: more variable and less variable. Fit a curve for each class and assess whether there are significant differences in subjects’ JND, slope, and reaction times across these trial classes. Investigate the effects of task conditions (Speaking, Listening, and Reading) and word congruency on these differences.

Given that this comment, too, provides suggestions for a separate study that seeks to investigate production variability (which was not assessed in our study) in relation to perceptual auditory skills, we refer to our previous responses above.

3. Exploring Practice Effects:

Further segregate trials into early (first 55 trials) and late (last 55 trials) categories to examine the effect of practice. Evaluate whether there are significant differences in subjects’ JND, slope, and reaction times between these two trial classes. Explore the potential impact of task conditions (Speaking, Listening, and Reading) and word congruency on these differences.

If any significant relationships are identified during these analyses, I encourage the authors to discuss their relevance in the manuscript. Such discussions can contribute to a deeper understanding of the study's implications and enhance the readers' appreciation of the research.

Following the reviewer’s suggestion, we attempted to implement the suggested analyses exploring additional practice effects on JND, slope, and reaction times (recall that in the paper we already explored the practice effect by analyzing whether formant discrimination improved across the 6 tasks performed during the entire session). This effort quickly revealed several obstacles to such an approach. First, if the trials were to be split up in early trials and late trials, all the logistic curve fitting would need to be re-done for those subsets of data separately and none of the data reported in the paper (based on logistic curve fitting of the full data sets) would be valid anymore. Second, even if one wanted to dismiss all the current data and analyses, doing the logistic curve fitting with greatly reduced data sets becomes problematic as the fitted functions then do not fit the raw data as well. Third, in fact, when we tried this approach, it became clear that we would need to eliminate another two participants from all analyses because their JND and/or slope could not be reliably estimated with only half as many trials. In the end, despite our best efforts to follow this suggestion, we had to conclude that going this route only introduced disadvantages without offering any major advantages, especially in light of the fact that our current analyses already include a different test of the practice effect.

For the reviewer’s information, we conducted some simple t-tests on the early vs. late split data of each task (thus without considering interactions with the Condition and Word List variables which would have led to the above described problems). These tests showed that there was no within-task practice effect for JND for any task. For slope, there were also no within-task practice effects for either speaking or listening; only for silent reading was the p value between .01 and .05. Thus, in the end, there was no within-task practice effect for the formant discrimination measures and thus no need to discuss the relevance of any such effect.

Minor comment: What do the authors mean by “entire experiment” in line 166: “The entire experiment consisted of 110 trials.” Do they mean each condition-word list combination? Please clarify this.

Thank you for pointing this out. We meant each condition-word list combination. We have changed the phrasing in line 166 to avoid confusion.

Reviewer #2: Manuscript: Perceptual formant discrimination during speech movement planning

This study examines the relationship between speech planning and auditory acuity. There has been previous work done on this, but specifically regarding loudness, which is potentially less relevant for speech motor control. The authors investigate formant acuity, which is potentially an aspect of speech motor control that is being actively predicted and planned during the planning period. The experiment tests acuity during speaking, listening, and reading conditions; in each condition, the vowel tested is either the same as what is produced (e.g. caret vowel tested, “mud” produced) or different (e.g. caret vowel tested, “mead” produced). Auditory stimuli are presented at a time that has previously been shown to be Participants had lower acuity in the speak condition compared to read, but not compared to listening; there was also no significant difference between listen and read. Furthermore, there was no effect of vowel. The authors conclude that this supports a general auditory suppression interpretation of PSAM, rather than an interpretation where modulation is driven by specific tuning of neural populations relevant to the motor task at hand.

Overall this is a well-thought out, well-written study, though with potentially a few caveats depending on further information. There are a few major points that I think should be addressed:

We thank the reviewer for this overall positive evaluation of the manuscript.

1. Clarity regarding methods. The authors state (L 166) “The entire experiment consisted of 110 trials”. In L 136 the authors refer to the “experiment” as having a pre-test and a series of speaking, listening, and silent reading tasks. So are the authors saying that there were only 110 trials for all 6 conditions? Or were there 110 trials in each condition, for a total of 660 trials? Was each wordlist read once with no repetitions? How many times was each vowel step heard under each condition*word list combination?

Thank you for pointing this out. Your second interpretation is correct, namely that there were 110 trials per condition, and thus 660 trials total across all conditions. We have changed the phrasing in line 166 to make this clear.

2. Power. The Merrikhi et al. 2018 paper only had 16 participants, and this one has 22 that were actually included. I would expect, and the authors seem to agree, that the effects being tested here are likely fairly small, especially the interaction with vowel quality of the spoken word. How did the authors arrive at 26 participants (with data loss to 22) as an appropriate sample size for this effect? In particular, the interpretation for PSAM as a general auditory modulation vs. specific modulation by neural population for the task hinges, as far as I can tell, on two differences that were not demonstrated here: 1. Statistically significant difference between listening and speaking; 2. Significant effect of word list.

We appreciate the reviewer’s’ comment. It reflects the intertwined nature of a number of complex issues. First, the reviewer refers to “the interpretation for PSAM as a general auditory modulation vs. specific modulation by neural population” and we would like to clarify the premise of the study. As we summarize in the opening paragraph of the Discussion, the original premise of the study was that formant discrimination during speech planning may help determine whether, at that time during speech planning, either specific auditory neuronal populations are suppressed (leading to reduced discrimination ability) or that specific patterns of combined suppression and facilitation may fine tune auditory cortex for better speech feedback monitoring (which could lead to enhanced discrimination ability for formant frequencies). We then described in the second and third paragraphs of the Discussion which results were obtained, and in paragraphs four and five that the results are consistent with (but with a few differences in the sense of fewer statistically significant effects despite the inclusion of more participants) a previous study by others. Hence, this led to the “Taken together, …” conclusion that, overall, the reduced discrimination findings are mostly in line with the suppression hypothesis. The reviewer’s comment made us realize that we should have clarified there that prior work on PSAM has demonstrated that the reduction in N1 amplitude occurs for auditory predictions associated with either speaking words or listening to prerecorded versions of one’s own productions (participants knew on each trial which word would be played). This was demonstrated in the Listening condition in Daliri & Max 2015a (which was already cited in our original submission) which includes the below figure. We have added a statement to this effect to the relevant section in the Discussion, and we believe that this discussion is now further improved.

Second, focusing on statistics and hypotheses, the above PSAM results from Daliri & Max (2015) already address the assumption that there should be a difference in formant discrimination between listening and speaking: that is not the case as PSAM may reflect a more general auditory prediction that occurs for both speaking and listening. Third, also related to statistics and hypotheses, the reviewer referred to an assumption that there should be a difference in formant discrimination before productions from the two word lists. With regard to this assumption, we would like to refer to the fact that PSAM has never been suggested to have that level of specificity: the basic PSAM phenomenon consists of the reduction, prior to speaking (as compared with silent reading), in the N1 response to a pure tone, thus not even a speech stimulus. Furthermore, it was demonstrated in Daliri & Max 2016 (which was already cited) that PSAM has the same effect on the response to a pure tone as on the response to a speech stimulus, as in the below figure from that paper. If the phenomenon affects the processing of a pure tone and a speech syllable in the same manner, it is more confirmatory rather than surprising that the present study found no effect of word list on formant discrimination at the same point in time. For increased clarity, we have added this important point in the revised Discussion section.

Less major points:

3. Alternative interpretation: This interpretation seems like it would predict task-specific effects, such as an effect of word list, which was not found here. If this is incorrect, can the authors provide a little more detail about the neural populations that would be recruited in feedforward planning for speech? It seems that they’d have to be broad—why?

It is not clear from the reviewer’s comment whether “alternative interpretation” refers to the Jenson & Saltuklaroglu (2021, 2022) interpretation of overlap in the networks involved in the planning for speech production and speech perception or our suggestion that a more narrow version could be proposed in which specifically auditory cortex is involved in the planning of speech movements (i.e., in feedforward control as opposed to its obvious involvement in feedback control). To the best of our knowledge, neither of these two interpretations/hypotheses would necessarily predict utterance-specific effects such as an effect of word list in our study. In the manuscript, we clearly state that the narrow hypothesis is a novel one; hence, at this point, it certainly isn’t developed at a level of detail that would allow speculation about which specific auditory neuronal populations may be involved in the feedforward planning of speech movements. We do, however, further clarify the novel hypothesis in our manuscript by suggesting that initial tests can examine whether quantitative measures of PSAM relate mostly to aspects of speech production known to reflect feedback control vs. those known to reflect feedforward control. As mentioned above, initial work along those lines (see our comment regarding the relationship between PSAM and inter-trial formant variability) is underway in our laboratory.

*Trivia:*

*L 37 has an extra space before the period*

*L 99 don’t understand the use of “already” in this context*

*L 176 currently “SLP” instead of “SPL”*

These mistakes have been corrected.
